# Factors Affecting Sick Leave Duration for Non-Work-Related Temporary Disabilities in Brazilian University Public Servants

**DOI:** 10.3390/ijerph15102127

**Published:** 2018-09-27

**Authors:** Adriano Dias, Juan Gómez-Salgado, João Marcos Bernardes, Carlos Ruiz-Frutos

**Affiliations:** 1 Public Health Grade Program, Botucatu Medical School, Paulista State University/UNESP, Botucatu, Sao Paulo 18618687, Brazil; dias.adriano@unesp.br (A.D.); jmbernardes@yahoo.com (J.M.B.); 2 Department of Nursing, Universidad de Huelva, 21007 Huelva, Spain; 3 Department of Sociology, Social Work and Public Health, Universidad de Huelva, 21007 Huelva, Spain; frutos@uhu.es; 4 Safety and Health Posgrade Program, Universidad Espíritu Santo, Samborondón (Guayaquil) 091650, Ecuador

**Keywords:** temporary, disability, sick leave, non-work-related illness, rehabilitation, work adaptation, public workers

## Abstract

Sickness absenteeism in public institutions compromises the execution of services, and may also generate direct impacts on the population that receives coverage. To determine if sick leave duration for temporary disabilities is associated with non-work-related illnesses (NWRI), a historical cohort study was carried out of workers at a Brazilian University. The Charlson Comorbidity Index (CCI) was obtained from the most prevalent diagnoses in each expert examination and from the corresponding days of sick leave per episode, adjusting simple and multiple Cox regression models. As a result, 70% of the NWRI temporary disabilities were due to depressive disorders, convalescence, and dorsalgia with a sick leave duration between 4 and 320 days. The factors of protection for sick leave durations until the rehabilitation were non-insulin-dependent diabetes mellitus and hypertension. Long-term sick leaves were observed in the cases that required rehabilitation of those workers diagnosed with recurrent depressive disorders, conjunctivitis, acute sinusitis, skin disorders, calculus of kidney and ureter, abdominal and pelvic pain, and same-level fall accidents. It is also worth noting that even in a disease that can justify long-term sick leaves, such as breast cancer, the duration may be shorter according to the worker’s capacity and self-efficacy.

## 1. Introduction

Sickness absenteeism, understood as the non-attendance at work due to a certified or licensed illness [1], is a known subject of concern among all institutions for its associated reduction of working capacity and productivity [2,3,4]. However, in public institutions, in addition to the cost increase inherent to the event, sick leaves compromise the execution of services, and may also generate direct impacts on the population that receives coverage.

Long-term sick leaves cause the return to work to be difficult, mainly when it involves rehabilitation, because it implies changes in positions, functions, and status depending on the limitations caused by the disease. Some factors hinder the adaptation to the new work, such as not feeling identified with oneself in another function, the reorganization of everyday activities according to new schedules and obligations, the creation of new bonds with co-workers, the fear of falling ill again, the need to accept the limits resulting from the disease, and the need to build a new professional identity [5], in addition to involving other personal, family, and social issues [6,7].

In Brazil, during 2015, almost two million sickness or accident benefits were granted [8]. In the state of São Paulo, from 2003 to 2006, nearly eight hundred thousand workers’ sick leaves were registered [3]. These data refer to specific circumstances covered by different insurance systems, which leads to difficulties when it comes to creating precise denominators. These circumstances, in turn, tend to be underestimated due to their severity and commitment. In addition, they thwart the attempt to establish the proportion of return-to-work cases that require a work adaptation.

A large proportion of the sick leaves are due to non-work-related illnesses (NWRI) causing temporary disabilities [9] (i.e., situations and diseases of non-occupational origin). The duration of the NWRI spells may vary according to different factors, ranging from worker sociodemographic characteristics, care services, working conditions, and activities developed by the company [10,11]. All these characteristics, which involve work and worker in a combined way, are reflected in the quality of the return-to-work period, if the return occurs (since a proportion of people cannot return to work).

Most epidemiological studies on NWRI are restricted to specific groups such as health workers [10,12]. Even more studies concern public workers, that is, workers with a mid-level education such as those in technical or administrative positions [13]. This leaves a large sector of the working population unexplored. This paper includes several university units involving different activity areas, which provides a broader view of sick leaves regarding workers in functions ranging from security and caretaking to teaching and research, thereby responding to this gap in the literature.

Long-term sick leaves are a concern for both the employer and the employee, as they have serious health, emotional, and economic consequences for individuals, companies, and the society in general. In light of this situation, return-to-work coordination programs have been designed and implemented. These programs include a variety of interventions, such as occupational therapy, physiotherapy, psychological therapy, medical interventions, workplace ergonomics, education, and social therapy. Despite the increasing popularity of such programs, their impact on workers’ health outcomes and cost-effectiveness is uncertain. Recent reviews and meta-analyses on return-to-work coordination programs have shown no benefits when compared to usual practice. Very low to moderate quality evidence suggests no benefits for return-to-work outcomes [14,15]. According to these results, it is necessary to continue investigating the factors that affect sick leaves and disabilities, in order to readjust programs and interventions that facilitate people’s return to work.

In some Brazilian public universities, sick leaves are followed up by general physicians (GPs), who are also university workers. This situation facilitates health revisions and may affect the return to work and rehabilitation process.

The objective of this study is to determine whether the duration of sick leaves due to non-work-related temporary disability, in this collective of public university workers, depends on them requiring or not an adaptation of their original work position, as well as to assess those variables that may have a greater influence.

## 2. Materials and Methods

An historical cohort study was carried out of the NWRI temporary disabilities of statutory workers on university campuses in the interior of the state of São Paulo, Brazil, between January 2010 and December 2015, authorized by the Human Resources managers of the five institutional units involved—four university units and one administrative unit—, and approved by the Botucatu Medical School Ethics Board (#1874625, 19 December 2016) in accordance with the Brazilian legislation on research with human beings (CEP/CONEP Resolution #196/96). An analysis was performed of all medical reports (census) from the institution’s own occupational medical service (called the Health Technical Section) that examines all statutory workers who request a sick leave with a duration of two days or more. This service, which manages all sick leaves and has the power of granting or denying them, is also responsible for the medical boards that decide on return-to-work cases. It is composed of general practitioners and psychiatrists.

For the database creation, information was extracted from two institutional databases: the Integrated Occupational Management (IOM) Software, which identifies the worker and stores data on the medical examination, and the Medical Care System (MCS), which records data about the rehabilitation and adaptation.

Once the information was integrated and treated, the database was built and it contained different types of variables. Among the sociodemographic variables were place of birth, sex, age at hiring and current age, marital status, and cohabitation with partner. In addition, functional characteristics were identified such as work position and unit, rehabilitation record, working time in the institution, and total working time. Other variables regarding the sick leave characteristics were analyzed: total duration of sick leaves, number of medical examinations carried out in the period, cause according to the International Classification of Diseases and Related Health Problems (ICD-10), and behavior among the ICD-10 chapters when the events were repeated. Finally, with respect to work adaptation, the following characteristics were found in the database: if there was an adaptation, the length of time until it occurred and the limitations. The reasons for each sick leave were defined as the first or the main cause indicated by the expert, since there could be more than one classification heading for each case.

**Figure 1:**
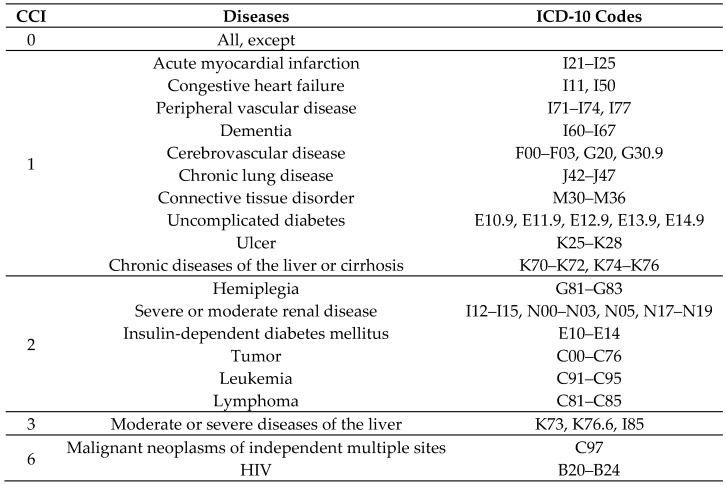
The Charlson Comorbidity Index distribution.

After the initial data treatment, the Charlson Comorbidity Index (CCI) [16] was introduced into the database, defined by 17 clinical conditions and adapted according to the ICD-10 codes, since the database had been created during the previous classification (CID-9), as shown in Figure 1 [17]. As for risk adjustment, the CCI defines clinical conditions that classify the severity of the case in order to adjust its effect on the response variable.

Exploratory data analysis was performed by percentage distributions of the most prevalent diseases or categories of each ICD-10 chapter for each medical examination conducted, as well as of the respective sick leave spell duration, including measures of central tendency and dispersion, and weights distribution according to the CCI.

The predictive sick leave variables were adjusted by a Cox regression model (univariate and multiple) for the response continuous variable ”sick leave duration”, the dichotomous status variable ”work adaptation” (which meant a return to work in a different condition, function, or activity type), and the covariates that were fully contemplated throughout the cohort: age at admission and working time (continuous), sex, presence of a partner, work unit and position, CCI, and most prevalent disease of each ICD-10 chapter (categorical), according to the literature [10,11,12,13]. The multiple Cox regression model was adjusted by stepwise insertion criteria and the maintenance of *p* < 0.05 in the final model. All analyses were carried out with the IBM/SPSS Statistics software, v.20.0, at a 5% significance level.

## 3. Results

Table 1 shows that among the 1753 cases of NWRI temporary disabilities obtained during the period, and for only 21 diseases chosen for being the most prevalent of the ICD-10 chapters, three of these diseases represented almost 70% of the sick leaves, of which 30% were depressive disorders (F33), followed by convalescence (Z54) and dorsalgia (M54) accounting for approximately 20% each. The proportions of sick leaves that had these diseases as main causes decreased according to the number of occurrences per worker, concentrating greater proportions of single (14%) or double spells (17%), reaching only approximately 2% between 13 and 20 spells of sick leaves.

Table 2 shows the measures for the central tendency and dispersion of the sick leave duration by episode of those same 21 diseases, being quite heterogeneous with each other, having an average duration from 4 to more than 320 days. Likewise, there was great variability between those sick leaves caused by the same disease, and great differences between the minimum and maximum sick leave duration, showing a range between 2 and 1439 days.

Some diseases presented the highest average duration of sick leaves, such as ”other congenital malformations of the spinal cord” (325.25 days on average), which affected four cases, the 36 malignant neoplasms of the breast (95.11 days on average), and the 42 cases of primary hypertension (92.55 days on average). When analyzing sick leaves with a high number of occurrences, we highlighted the 499 recurrent depressive disorders (58.55 days on average), the 367 convalescences (37.55 days on average), and the 334 cases of dorsalgia (31.54 days on average).

The distribution of diseases using the Charlson Comorbidity Index produced 1687 events classified as weight 0 (96.2%), 30 (1.7%) as 1, and 36 (2.1%) as 2. There were no records with weights 3 or 6 among the most prevalent diseases attributed as a cause of sick leave (Figure 1).

Table 3 presents the adjustments of the Cox simple regression model to determine the association of covariates and the time until rehabilitation by means of hazard ratios (HR) and their respective confidence intervals at 95%. From this table, we can highlight that “live with a partner” HR: 0.85 (0.74–0.98) and work at the “Agricultural sciences” unit HR: 0.63 (0.47–0.85) were independently associated to sick leave duration for the workers that returned to work in a different type of activity.

An a priori defined criterion was not established. In its place, the decision was made to adjust a multiple Cox model with all the variables used in the simple adjustments in order to control possible unknown confusion and collinearity variables between them, which resulted in a quite different outcome from the one involving simple adjustments. The results are shown in Table 4, where they remain statistically significant in the final model, as a factor of protection for the time until rehabilitation, the number of medical examinations HR = 0.96 (0.95–0.96), as well as non-insulin-dependent diabetes mellitus HR = 0.40 (0.17–0.95) and primary hypertension HR = 0.29 (0.15–0.55).

On the other hand, when the data were compared with those for infectious or parasitic diseases, which are used as a reference category, we found increased probabilities of long-term sick leaves in the cases that required the rehabilitation of the workers diagnosed with recurrent depressive disorders HR = 1.5 (1.18–1.94), conjunctivitis HR = 2.78 (1.96–3.94), acute sinusitis HR = 4.99 (2.68–9.30), skin conditions HR = 3.80 (2.15–6.72), dorsalgia HR = 1.62 (1.25–2.10), calculus of kidney and ureter HR = 2.31 (1.27–4.20), abdominal and pelvic pain HR = 2.33 (1.23–4.41), and falls on the same level HR = 3.71 (1.15–11.97).

## 4. Discussion

In this study, we were able to identify variables that differentiate sick leave duration data according to a required work adaptation, or not, in a wider collective than the one usually found in the literature, that is, education and health professionals. Although the latter are linked to a public university, the institution also includes non-educational collectives.

From previous studies, we obtained data on the high cost of sick leaves, including the effects on the workers involved, the entrepreneurs and/or the society in general. Job factors played an important role in this relationship, as well as the fact that effective preventive measures were in place to reduce sick leaves such as the gradual return to work, that reduces the cases of permanent disability [18].

We observed that the more medical consultations were made, the less likely longer sick leave times were needed for those workers who required a rehabilitation (HR < 1), something that we had not previously found in the literature. We can consider whether a more continuous follow-up of cases makes it easier to reduce sick leave duration in those cases that will eventually need work adaptation.

Among the causes of sick leave with a need for rehabilitation, we found it difficult to act in two of the three causes with a longer average sick leave duration. The disease with the greatest average duration of the sick leave/return, under the heading “Other congenital spinal cord malformations” (325 days on average), presented only four cases and a pathology of difficult treatment. The next disease with a greater average duration (95 days) was a serious disease (malignant neoplasms of the breast) and, in this case, the number was certainly higher (36 cases), although it would seem logical for the disease to imply a greater sick leave time. From the literature, we know that two years after the diagnosis of breast cancer, between 13% and 40% of women had not yet returned to work [19,20]. However, it has also been claimed that there are factors, such as working capacity or self-efficacy, which are the key predictors for an early return to work in cases of cancer diagnoses [21].

For the third type of disease in order of occurrence, “primary hypertension”, with an average sick leave duration of 92 days (8.50 of median) and 42 cases, we could in fact assess whether it was a suitable time lapse as compared with other studies. We could also take this into account to reduce work adaptation times in such cases. In their 2012 paper, Cabanillas et al. [22] found that the median duration of the sick leave for primary hypertension in the Autonomous Community of Andalusia (Southern Spain) was 14 days, although this fact was not obtained from a group of workers, but from the general population.

If we analyze the three sick leave causes with a greater number of cases, one cause falls under the hardly informative heading “convalescence” (367 cases and 37.55 days of sick leave on average). It encompasses a large number of possible pathologies, and the only useful factor in such cases would be to have additional information available to make it easier to determine if the sick leave or return-to-work times were adequate. Another cause of a high number of sick leaves was found under the heading “recurrent depressive disorders” (500 cases and 58.55 days of sick leave on average), where not only the number of cases was high, but also the average duration of the sick leave. This is consistent with the literature, which claims that, on a global scale, mental conditions in general, and depression in particular, generate a high percentage of sick leaves and they are long-term [23]. The trend to prolong sick leaves in some diagnoses such as psychiatric diseases has been shown in previous studies [24]. An historical cohort study from Scotland, with more than 50,000 spells of sick leaves, showed that the median duration of sick leaves for depressive disorders was 54 days [25]. In a study carried out in Spain, the median duration of sick leaves caused by unspecified anxiety states was 25 days, and by neurotic depression, 52 days [21]. Dewa et al. [26], in their 2014 systematic review of sick leaves due to mental disorders, found median times of sick leaves between 5 and 119 days. The third cause with a high number of cases was “dorsalgia” (334 cases, 31.54 days on average and a median of 10 days of sick leave). Musculoskeletal disorders were a very common cause of sick leave, as described in the literature, particularly spine (neck or back pain) and upper limb disorders [27,28]. Specifically, we found an average time of 43.5 days and a median of 10 days for sick leaves due to musculoskeletal disorders, showing low-back pain and neck pain the shortest ones, with a median of 7 days [25]. A study carried out in Norway on the musculoskeletal conditions of workers found that the mean length of absence due to sickness was 101 days for back disorders, 110 days for upper limb conditions, and 164 days for osteoarthrosis [28].

Because of the magnitude of the two last causes of sick leaves, it would be interesting to find the most efficient way to manage them, because of both the personal and economic benefits that would occur due to the reduction of their duration.

It may seem logical that the duration of sick leaves with a need for work adaptation (Table 4) would be lower than that of intestinal infectious diseases, which serve as a reference, in those cases diagnosed with non-insulin-dependent diabetes mellitus and primary hypertension, because they are well-diagnosed pathologies which require frequent health care. The same situation occurs with the opposite case, where sick leave times prior to work adaptation are higher in diagnoses such as recurrent depressive disorders, dorsalgia, abdominal and pelvic pain, or calculus of kidney and ureter. However, we did not find a straightforward explanation for including in the same group the cases diagnosed with acute sinusitis, falls on the same level, or conjunctivitis, due to the specific character of these minor pathologies that have a short duration and are associated with simple treatments.

The strengths of this study included the sample characteristics, the large number of cases included in the study, and the wide variability of educational levels and professions of the study population. Another strength of our study was the surveillance system used for the sick leaves, that is, the surveillance carried out by the same group of doctors belonging to the company-university. This allowed us to analyze the “number of consultations” variable in a reliable way.

It is necessary to emphasize that our study had a limitation: in the analyzed databases, we found workers who were on sick leave at the start of the study and continued being on sick leave 1439 days later, which is when the study period ended, because there was no resolution to their professional activity and ability. Therefore, the sick leave time range was between 2 and 1439 days, although it could have been higher. Another important limitation was related to the decision to use the first of the records as the primary cause of sick leaves, since a significant proportion of the workers presented more than one reason (or complaint) and the first cause may not have been the main one for the sick leave, making it impossible to identify it through secondary data. This may, in part, explain the long-term sick leaves associated with some minor diseases and of presumably short duration.

Future lines of research could consider developing an algorithm that considers age, sex, diagnosis of sick leave, and comorbidity, in order to calculate the optimal sick leave duration at the individual level, as has been published about the general population [22]. This information would be greatly improved by including the job position, as it is highly predictive of the sick leave duration and is easy to obtain in large companies like that of this study, and by applying it to the cases requiring work adaptation as well as to those which do not.

## 5. Conclusions

In our study, we identified variables associated with a greater likelihood of extending sick leave duration in cases requiring an adaptation of the job position. Considering our results, we suggest that it would be necessary to act upon cases of hypertension because of the number of sick leaves associated with it, taking into account however that sick leave duration is not higher in those cases that involve a work adaptation than those that do not require it. It is also worth highlighting the fact that pathologies have been identified with long periods of sick leave, such as the case of cancer where, although the sick leaves are largely justified, interventions could be implemented to reduce the duration. Among the factors in our study that may require further research, we found pathologies that have shown a difference between sick leave times regarding required work adaptation or not, such as recurrent depressive disorders, dorsalgia, abdominal and pelvic pain, and calculus of kidney and ureter. In addition, results showed that the increase of consultations during the period of sick leave reduced its duration in those people who required a work adaptation. The management of workers’ return to work is a very complex strategy that involves the roles of multiple factors beyond the disease. It is difficult to know if any of these factors are similar among all conditions and settings, and whether it is possible to draw and apply a generic strategy to improve the quality of job reinsertion and to reduce times. The findings from this study could guide policies and interventions regarding the management of sick leaves and disabilities.

## Figures and Tables

**Table 1:** Most prevalent diseases per ICD-10 chapter, in the first 20 medical examinations performed.

		Examination																					
Chapter	Disease	1	2	3	4	5	6	7	8	9	10	11	12	13	14	15	16	17	18	19	20	Total	%
I	A09	8	5	6	1	1	1	0	1	3	0	0	0	1	0	0	0	2	0	1	0	30	1.71
II	C50	9	7	6	2	3	2	2	2	0	1	0	1	0	1	0	0	0	0	0	0	36	2.05
III	D69	1	0	0	0	0	0	1	1	1	1	1	0	0	0	0	0	0	0	0	0	6	0.34
IV	E11	7	2	1	2	3	3	2	1	1	1	1	1	0	0	1	1	0	2	0	1	30	1.71
V	F33	38	42	42	33	25	27	32	26	28	25	22	26	22	19	23	16	16	13	12	12	499	28.47
VI	G56	8	3	3	4	2	2	1	1	0	0	1	0	1	0	0	2	2	0	1	1	32	1.83
VII	H10	56	37	13	10	6	5	6	3	0	1	1	4	6	3	2	1	0	2	0	1	157	8.96
VIII	H81	3	2	1	1	5	0	1	0	1	0	1	0	1	0	1	1	1	0	1	0	20	1.14
IX	I10	13	6	8	2	3	2	1	0	1	1	0	0	1	2	1	0	1	0	0	0	42	2.40
X	J01	4	3	2	2	1	1	3	3	0	0	2	0	1	1	1	0	0	0	0	0	24	1.37
XI	K80	13	2	1	0	1	0	0	0	0	0	0	0	0	2	0	1	0	0	0	0	20	1.14
XII	L98	1	2	4	2	2	0	0	0	2	1	2	1	0	3	1	1	0	1	0	0	23	1.31
XIII	M54	52	39	30	27	24	22	12	15	18	8	15	13	7	12	10	8	7	2	4	9	334	19.05
XIV	N20	9	8	6	3	0	1	2	1	1	1	0	0	0	1	2	0	1	1	2	1	40	2.28
XV	O47	0	2	0	3	1	0	0	0	0	0	0	0	0	0	0	0	0	0	0	0	6	0.34
XVI	P05	1	0	0	0	0	0	0	0	0	0	0	0	0	0	0	0	0	0	0	0	1	0.06
XVII	Q06	0	0	0	0	1	0	1	0	0	1	0	1	0	0	0	0	0	0	0	0	4	0.23
XVIII	R10	3	2	2	2	1	1	1	0	0	1	2	4	1	1	1	0	0	0	0	1	23	1.31
XIX	S82	9	11	8	6	5	3	2	1	0	1	1	2	0	1	1	1	0	2	1	0	55	3.14
XX	W01	0	1	0	1	0	0	1	0	0	0	0	0	1	0	0	0	0	0	0	0	4	0.23
XXI	Z54	4	121	42	52	19	20	18	16	13	14	12	5	6	4	4	4	3	3	2	5	367	20.94
	Total	239	295	175	153	103	90	86	71	69	57	61	58	48	50	48	36	33	26	24	31	1753	
	%	13.63	16.83	9.98	8.73	5.88	5.13	4.91	4.05	3.94	3.25	3.48	3.31	2.74	2.85	2.74	2.05	1.88	1.48	1.37	1.77		100.0

A09 (Other gastroenteritis and colitis of infectious and unspecified origin), C50 (Malignant neoplasm of breast), D69 (Purpura and other hemorrhagic conditions), E11 (Non-insulin-dependent diabetes mellitus), F33 (Recurrent depressive disorder), G56 (Mononeuropathies of upper limb), H10 (Conjunctivitis), H81 (Disorders of vestibular function), I10 (Essential (primary) hypertension), J01 (Acute sinusitis), K80 (Cholelithiasis), L98 (Other disorders of skin and subcutaneous tissue, not elsewhere classified), M54 (Dorsalgia), N20 (Calculus of kidney and ureter), O47 (False labor), P05 (Slow fetal growth and fetal malnutrition), Q06 (Other congenital malformations of spinal cord), R10 (Abdominal and pelvic pain), S82 (Fracture of lower leg, including ankle), W01 (Fall on same level from slipping, tripping and stumbling), Z54 (Convalescence).

**Table 2:** Sick leave duration distribution of the 21 most prevalent diseases per ICD-10 chapter, in the first 20 expert examinations performed.

	n	mean	SD	p25	p50	p75	Minimum	Maximum
A09	30	3.83	2.49	2.00	3.00	5.00	2	12
C50	36	95.11	146.82	30.00	90.00	90.00	9	729
D69	6	18.33	17.01	7.00	13.50	21.00	5	50
E11	30	45.20	28.10	17.00	47.00	60.00	2	90
F33	499	58.55	119.23	30.00	51.00	60.00	2	1439
G56	32	36.72	92.06	10.00	15.00	30.00	2	533
H10	157	5.93	2.67	5.00	5.00	7.00	2	15
H81	20	17.15	14.46	5.00	11.00	30.00	2	45
I10	42	92.55	273.09	4.00	8.50	30.00	2	1275
J01	24	4.75	3.73	3.00	4.00	5.00	2	20
K80	20	13.95	11.06	4.00	13.00	17.50	2	40
L98	23	12.13	13.86	4.00	7.00	14.00	2	60
M54	334	31.54	103.57	5.00	10.00	30.00	2	927
N20	40	8.20	8.67	2.50	5.00	9.00	2	30
O47	6	12.17	4.22	10.00	10.00	14.00	9	20
P05	1	7.00	--	7.00	7.00	7.00	7	7
Q06	4	325.25	511.28	60.00	90.00	590.50	30	1091
R10	23	8.57	7.98	4.00	5.00	10.00	2	30
S82	55	51.64	30.50	28.00	45.00	90.00	2	91
W01	4	5.75	3.50	3.00	5.50	8.50	2	10
Z54	367	37.55	116.57	10.00	17.00	30.00	2	1439

SD: standard deviation; p25: percentile 25; p50; percentile 50; p75: percentile 75. A09 (Other gastroenteritis and colitis of infectious and unspecified origin), C50 (Malignant neoplasm of breast), D69 (Purpura and other hemorrhagic conditions), E11 (Non-insulin-dependent diabetes mellitus), F33 (Recurrent depressive disorder), G56 (Mononeuropathies of upper limb), H10 (Conjunctivitis), H81 (Disorders of vestibular function), I10 (Essential (primary) hypertension), J01 (Acute sinusitis), K80 (Cholelithiasis), L98 (Other disorders of skin and subcutaneous tissue, not elsewhere classified), M54 (Dorsalgia), N20 (Calculus of kidney and ureter), O47 (False labor), P05 (Slow fetal growth and fetal malnutrition), Q06 (Other congenital malformations of spinal cord), R10 (Abdominal and pelvic pain), S82 (Fracture of lower leg, including ankle), W01 (Fall on same level from slipping, tripping and stumbling), Z54 (Convalescence).

**Table 3:** Adjustments of the Cox multiple regression model, by hazard ratio, confidence intervals, and *p* values, for the duration of university workers’ sick leaves requiring work adaptation, between 2010 and 2015.

	HR	95.0% CI for HR		*p*-Value
		Lower	Upper	
Sex	1.046	0.960	1.139	0.303
Age at the start of the process	0.992	0.981	1.004	0.195
Time working at university	1.003	0.992	1.014	0.619
Lives with a partner	0.849	0.736	0.981	0.026
Unit				
Administration				
Agricultural sciences	0.632	0.472	0.846	0.002
Human health	0.901	0.661	1.228	0.209
Animal health	1.051	0.897	1.230	0.241
Biological sciences	1.278	0.875	1.865	0.204
Position				
Administration				
Field	4.003	0.000	---	0.914
Teaching	9.254	0.000	---	0.863
Health (middle level)	0.685	0.000	---	0.977
Operational	13.789	0.000	---	0.839
Others (upper level)	9.752	0.000	---	0.860
Others (high level)	11.550	0.000	---	0.850
Radiotherapy	0.000	0.000	---	0.926
Health (upper level)	10.674	0.000	---	0.855
Supervisory	3.182	0.000	---	0.929
Academic support	2.820	0.000	---	0.936
Transport	4.235	0.000	---	0.911
Security and reception	0.000	0.000	---	0.943
Disease				
A09	1	---	---	---
C50	1.389	0.073	5.434	0.227
D69	0.938	0.049	4.987	0.166
E11	0.291	0.007	3.508	0.211
F33	0.374	0.019	4.289	0.116
G56	0.680	0.037	6.357	0.235
H10	1.155	0.061	7.828	0.223
H81	2.744	0.150	10.093	0.196
I10	0.687	0.036	7.156	0.203
J01	0.349	0.018	4.586	0.242
K80	6.250	0.337	45.811	0.219
L98	1.647	0.075	5.072	0.249
M54	3.569	0.192	20.467	0.194
N20	0.940	0.052	6.083	0.236
O47	1.993	0.106	15.569	0.245
P05	1.513	0.063	13.472	0.199
Q06	0.325	0.000	7.466	0.239
R10	0.259	0.012	3.667	0.191
S82	1.103	0.057	4.172	0.248
W01	0.506	0.027	4.441	0.238
Z54	4.258	0.210	25.534	0.246
Charlson Comorbidity Index	0.852	0.633	1.146	0.289
Number of medical exams performed	0.984	0.959	1.041	0.243

HR: Hazard ratio. A09 (Other gastroenteritis and colitis of infectious and unspecified origin), C50 (Malignant neoplasm of breast), D69 (Purpura and other hemorrhagic conditions), E11 (Non-insulin-dependent diabetes mellitus), F33 (Recurrent depressive disorder), G56 (Mononeuropathies of upper limb), H10 (Conjunctivitis), H81 (Disorders of vestibular function), I10 (Essential (primary) hypertension), J01 (Acute sinusitis), K80 (Cholelithiasis), L98 (Other disorders of skin and subcutaneous tissue, not elsewhere classified), M54 (Dorsalgia), N20 (Calculus of kidney and ureter), O47 (False labor), P05 (Slow fetal growth and fetal malnutrition), Q06 (Other congenital malformations of spinal cord), R10 (Abdominal and pelvic pain), S82 (Fracture of lower leg, including ankle), W01 (Fall on same level from slipping, tripping and stumbling), Z54 (Convalescence).

**Table 4:** Adjustments of the Cox multiple regression model, by hazard ratio, confidence intervals, and *p* values, for the duration of university workers’ sick leaves requiring work adaptation, between 2010 and 2015.

	Parameter Estimate	Standard Error	Chi-Squared	HR	95.0% CI for HR		*p*-Value
					Lower	Upper	
Number of medical exams performed	−0.044	0.004	107.809	0.957	0.949	0.965	<0.001
Disease							
A09 (Other gastroenteritis and colitis of infectious and unspecified origin)	---	---	---	1	---	---	---
E11 (Non-insulin-dependent diabetes mellitus)	−0.908	0.439	4.275	0.403	0.170	0.954	0.038
F33 (Recurrent depressive disorder)	0.414	0.127	10.523	1.514	1.178	1.945	0.001
H10 (Conjunctivitis)	1.022	0.177	33.198	2.780	1.963	3.937	<0.001
I10 (Essential (primary) hypertension)	−1.259	0.337	13.945	0.284	0.147	0.550	<0.001
J01 (Acute sinusitis)	1.608	0.317	25.625	4.993	2.679	9.306	<0.001
L98 (Other disorders of skin and subcutaneous tissue, not elsewhere classified)	1.335	0.290	21.111	3.802	2.151	6.721	<0.001
M54 (Dorsalgia)	0.484	0.131	13.543	1.623	1.254	2.100	<0.001
N20 (Calculus of kidney and ureter)	0.839	0.304	7.597	2.315	1.274	4.205	<0.001
R10 (Abdominal and pelvic pain)	0.844	0.326	6.689	2.328	1.227	4.415	<0.001
W01 (Fall on same level from slipping, tripping and stumbling)	1.310	0.597	4.809	3.710	1.150	11.972	0.028

HR: Hazard ratio.
